# *International Journal of Environmental Research and Public Health* Best Paper Award 2015

**DOI:** 10.3390/ijerph120101050

**Published:** 2015-01-20

**Authors:** Paul B. Tchounwou, Joyce Zhou

**Affiliations:** 1 Molecular Toxicology Research Laboratory, Jackson State University, 1400 Lynch Street, Box 18750, Jackson, MS 39217, USA; E-Mail: paul.b.tchounwou@jsums.edu; 2 MDPI AG, Klybeckstrasse, CH-4057 Basel, Switzerland and MDPI Branch Office, Beijing 101101, China; E-Mail: joyce.zhou@mdpi.com

*International Journal of Environmental Research and Public Health* started to institute an annual award in 2013 to recognize outstanding papers related to environmental health sciences and public health that meet the aims, scope and high standards of this journal. We are pleased to announce the third “*International Journal of Environmental Research and Public Health* Best Paper Awards” for 2015. Nominations were solicited from the Editor-in-Chief and Editorial Board members, with all papers published in 2011 eligible for consideration. The following nine papers were selected for recognition awards:

**Article Award:**
  ***1^st^ Prize***
    **Authors: Sid E. O’Bryant, Melissa Edwards, Chloe V. Menon, Gordon Gong and Robert Barber**
    Article title: Long-Term Low-Level Arsenic Exposure Is Associated with Poorer Neuropsychological Functioning: A Project FRONTIER Study
    *Int. J. Environ. Res. Public Health* **2011**, *8*(3), 861–874; doi:10.3390/ijerph8030861
    Available online: http://www.mdpi.com/1660-4601/8/3/861
  ***2^nd^ Prize***
    **Authors: Gert S. Maritz and Richard Harding**
    Article title: Life-long Programming Implications of Exposure to Tobacco Smoking and Nicotine before and soon after Birth: Evidence for Altered Lung Development
    *Int. J. Environ. Res. Public Health* **2011**, *8*(3), 875–898; doi:10.3390/ijerph8030875
    Available online: http://www.mdpi.com/1660-4601/8/3/875
  ***3^rd^ Prize***
    **Authors: Reinskje Talhout, Thomas Schulz, Ewa Florek, Jan Van Benthem, Piet Wester and Antoon Opperhuizen**
    Article title: Hazardous Compounds in Tobacco Smoke
    *Int. J. Environ. Res. Public Health* **2011**, *8*(2), 613–628; doi:10.3390/ijerph8020613
    Available online: http://www.mdpi.com/1660-4601/8/2/613
  ***4^th^ Prize***
    **Authors: Dustin T. Duncan, Jared Aldstadt, John Whalen, Steven J. Melly and Steven L. Gortmaker**
    Article title: Validation of Walk Score^®^ for Estimating Neighborhood Walkability: An Analysis of Four US Metropolitan Areas
    *Int. J. Environ. Res. Public Health* **2011**, *8*(11), 4160–4179; doi:10.3390/ijerph8114160
    Available online: http://www.mdpi.com/1660-4601/8/11/4160
  ***5^th^ Prize***
    **Authors: Richard F. Hector, George W. Rutherford, Clarisse A. Tsang, Laura M. Erhart, Orion McCotter, Shoana M. Anderson, Kenneth Komatsu, Farzaneh Tabnak, Duc J. Vugia, Ying Yang and John N. Galgiani**
    Article title: The Public Health Impact of Coccidioidomycosis in Arizona and California
    *Int. J. Environ. Res. Public Health* **2011**, *8*(4), 1150–1173; doi:10.3390/ijerph8041150
    Available online: http://www.mdpi.com/1660-4601/8/4/1150
**Review Award:**
  ***1^st^ Prize***
    **Authors: Christos A. Damalas and Ilias G. Eleftherohorinos**
    Article title: Pesticide Exposure, Safety Issues, and Risk Assessment Indicators
    *Int. J. Environ. Res. Public Health* **2011**, *8*(5), 1402–1419; doi:10.3390/ijerph8051402
    Available online: http://www.mdpi.com/1660-4601/8/5/1402
  ***2^nd^ Prize***
    **Authors: Daria J. Kuss and Mark D. Griffiths**
    Article title: Online Social Networking and Addiction—A Review of the Psychological Literature
    *Int. J. Environ. Res. Public Health* **2011**, *8*(9), 3528–3552; doi:10.3390/ijerph8093528
    Available online: http://www.mdpi.com/1660-4601/8/9/3528
  **Authors: Dianne Lowe, Kristie L. Ebi and Bertil Forsberg**
    Article title: Heatwave Early Warning Systems and Adaptation Advice to Reduce Human Health Consequences of Heatwaves
    *Int. J. Environ. Res. Public Health* **2011**, *8*(12), 4623–4648; doi:10.3390/ijerph8124623
    Available online: http://www.mdpi.com/1660-4601/8/12/4623
  ***3^rd^ Prize***
    **Authors: Andrew D. Kraft and G. Jean Harry**
    Article title: Features of Microglia and Neuroinflammation Relevant to Environmental Exposure and Neurotoxicity
    *Int. J. Environ. Res. Public Health* **2011**, *8*(7), 2980–3018; doi:10.3390/ijerph8072980
    Available online: http://www.mdpi.com/1660-4601/8/7/2980

The prize awarding committee recognizes the article “Long-Term Low-Level Arsenic Exposure Is Associated with Poorer Neuropsychological Functioning: A Project FRONTIER Study” as a “…very well designed and hypothesis-driven study that shows the association between low-level arsenic exposure and neuropsychological functioning. The reported findings are consistent with prior work linking environmental exposure to arsenic to poorer neuropsychological functioning…”. The article “Life-long Programming Implications of Exposure to Tobacco Smoking and Nicotine before and soon after Birth: Evidence for Altered Lung Development” offers “…some new insights into the emerging field of Developmental Origins of Adult Health and Disease (DoHAD), with regard to the development of lung disease induced by maternal cigarette smoking. The implication of epigenetic changes associated with disease etiology is highlighted in this paper…”. The article “Hazardous Compounds in Tobacco Smoke” provides “…a comprehensive list of hazardous smoke components, and may serve as a valuable resource for progressive reduction in the level of chemicals in tobacco smoke…”. The article “Pesticide Exposure, Safety Issues and Risk Assessment Indicators” comprehensively reviews “…pesticide toxicity, exposure evaluation, and public health risk assessment and management; and recommends strategies to protect public health as well as the environment…”. The article “Online Social Networking and Addiction—A Review of the Psychological Literature” reviews “…emerging phenomenon of addiction to Social Networking Sites (SNSs), adding some new aspects in this scarce research domain with focus on SNS addiction specifically and comorbidities...”. The article “Heatwave Early Warning Systems and Adaptation Advice to Reduce Human Health Consequences of Heatwaves” is an excellent review on “…particular measures to increase resilience to heatwaves, which will be useful for improving and enhancing current action plans…”. The article “Features of Microglia and Neuroinflammation Relevant to Environmental Exposure and Neurotoxicity” offers “… a new insight into the neuro network regulating cytokine release and inflammation pathways in environmental diseases and dissects whether the inflammation is a cause or result of the insult…”.

These nine exceptional papers are all valuable contributions to the *International Journal of Environmental Research and Public Health* and the public health field. On behalf of the Prize Awarding Committee and the Editorial Board of *International Journal of Environmental Research and Public Health*, we would like to congratulate the authors and co-authors of these nine papers for their excellent work. In recognition of their accomplishments, Drs. Sid E. O’Bryant, Richard Harding, Reinskje Talhout, Dustin T. Duncan and Richard F. Hector will prizes of 1,000 CHF, 800 CHF, 600 CHF, 400 CHF and 200 CHF, respectively. They will also have the privilege of publishing an additional paper free of charge in open access format in the *International Journal of Environmental Research and Public Health*, after normal peer-review. Drs. Christos A. Damalas, Daria J. Kuss, Dianne Lowe, and G. Jean Harry will be awarded the privilege of publishing an additional research paper free of charge in open access format in the *International Journal of Environmental Research and Public Health*, after normal peer-review. 

Prize Awarding Committee

Editor-in-Chief

**Prof. Dr. Paul B. Tchounwou**

Molecular Toxicology Research Laboratory, Jackson State University, 1400 Lynch Street, Box 18750, Jackson, MS 39217, USA

E-Mail: paul.b.tchounwou@jsums.edu

Editorial Board Member

**Prof. Dr. Assaf A. Abdelghani**

Department of Environmental Health Sciences, Tulane Universiaty School of Public Health of Tropical Medicine, New Orleans, LA 70112, USA

E-Mail: assafa@tulane.edu

Editorial Board Member and Guest Editor

**Prof. Dr. Emmanuel Rudatsikira**

School of Health Professions, Andrews University, Berrien Springs, MI 49104, USA

E-Mail: rudatsikira@andrews.edu

Editorial Board Member

**Prof. Dr. William A. Toscano**

Division of Environmental Health Sciences, School of Public Health, University of Minnesota, Minneapolis, MN 55455, USA

E-Mail: tosca001@umn.edu

Managing Editor

**Dr. Joyce Zhou**

MDPI Beijing Office, Suite 2011, Ruidu International Center, No. 1 Cuijingbeili, Tongzhou District, Beijing 101101, China

E-Mail: joyce.zhou@mdpi.com

